# Correlating Genotype and Phenotype in the Asexual Yeast *Candida orthopsilosis* Implicates *ZCF29* in Sensitivity to Caffeine

**DOI:** 10.1534/g3.119.400348

**Published:** 2019-07-27

**Authors:** Kontxi Martinez de San Vicente, Markus S. Schröder, Lisa Lombardi, Elise Iracane, Geraldine Butler

**Affiliations:** School of Biomolecular and Biomedical Science, Conway Institute, University College Dublin, Belfield, Dublin 4, Ireland

**Keywords:** *Candida*, genotype, pathogenic, phenotype, yeast

## Abstract

*Candida orthopsilosis* is diploid asexual yeast that causes human disease. Most *C. orthopsilosis* isolates arose from at least four separate hybridizations between related, but not identical, parents. Here, we used population genomics data to correlate genotypic and phenotypic variation in 28 *C. orthopsilosis* isolates. We used cosine similarity scores to identify 65 variants with potential high-impact (deleterious effects) that correlated with specific phenotypes. Of these, 19 were Single Nucleotide Polymorphisms (SNPs) that changed stop or start codons, or splice sites. One variant resulted in a premature stop codon in both alleles of the gene *ZCF29* in *C. orthopsilosis* isolate 185, which correlated with sensitivity to nystatin and caffeine. We used CRISPR-Cas9 editing to introduce this polymorphism into two resistant *C. orthopsilosis* isolates. Introducing the stop codon resulted in sensitivity to caffeine and to ketoconazole, but not to nystatin. Our analysis shows that it is possible to associate genomic variants with phenotype in asexual *Candida* species, but that only a small amount of genomic variation can be easily explored.

Population genomics studies of fungi are increasing, ranging from small-scale studies (approximately 20 isolates) to extremely large-scale studies (>1000 isolates) (Talas *et al.* 2016; Taylor *et al.* 2017; Peter *et al.* 2018; Ropars *et al.* 2018). These studies have revealed the high level of genetic variation among isolates, and sometimes uncovered evidence of distinct populations (Liti *et al.* 2009; Ellison *et al.* 2011; Schröder *et al.* 2016; Cissé *et al.* 2018; Ropars *et al.* 2018).

The association of phenotype with genetic variation can be studied in a number of ways, including linkage analysis and characterization of Quantitative Trait Loci (QTL), population genomics, and Genome Wide Association Studies (GWAS). All these approaches have been successfully applied in fungi, for example to identify loci associated with virulence, stress response and metabolism (Taylor *et al.* 2017). In *Saccharomyces* species QTL have been identified that correlate with response to stress (Kvitek *et al.* 2008), sugar transport (Bergström *et al.* 2014), xylose metabolism (Wenger *et al.* 2010) and adaptation to low temperature (García-Ríos *et al.* 2017), among others (Liti *et al.* 2017). QTL analysis has been used successfully in other fungi also, including *Lachancea kluyveri* (Sigwalt *et al.* 2016), *Neurospora* (Kim *et al.* 2007) and plant fungal pathogens (Lendenmann *et al.* 2016). GWA studies identified polymorphisms associated with virulence in fungal pathogens of plants and animals (Dalman *et al.* 2013; Talas *et al.* 2016; Zhong *et al.* 2017; Desjardins *et al.* 2017). A very large population genomics study of 1,011 *S. cerevisiae* isolates identified 35 variants associated with 14 conditions (Peter *et al.* 2018).

Standard linkage analysis cannot be applied in many fungal pathogens of humans, such as some in the *Candida* clade, where the species are asexual or have very rare, cryptic, sexual cycles (Wolfe and Butler 2017). Some studies however have identified causative variants in populations. For example, a population genomics study of 21 non-sexual *C. albicans* isolates identified a genetic variant causing a premature stop codon in the transcription factor *EFG1* that is correlated with decreased virulence (Hirakawa *et al.* 2015). Ropars *et al.* (Ropars *et al.* 2018) speculated that the presence of stop codons in transcription factor genes in one clade of *C. albicans* is associated with reduced virulence. However, there is a lack of systematic analyses of the contribution of individual genes to phenotype in pathogenic *Candida* species.

*C. albicans* belongs to the Lodderomyces group of the CTG-Ser clade, in which the CUG codon is translated as serine rather than leucine (Santos and Tuite 1995). Most (and perhaps all) members of the Lodderomyces clade have diploid genomes and do not undergo meiosis. The Lodderomyces clade includes the *Candida parapsilosis sensu lato* species group - C. *parapsilosis*, *Candida orthopsilosis* and *Candida metapsilosis* (Tavanti *et al.* 2005). Mating or meiosis have never been observed in *C. parapsilosis sensu lato* (Sai *et al.* 2011). *C. parapsilosis* is the most commonly isolated pathogen from this group, and it is of particular concern in very low weight neonates (Chow *et al.* 2012). *C. orthopsilosis* is second or third depending on the geographical region (Cantón *et al.* 2011; Bonfietti *et al.* 2012; Chen *et al.* 2015).

*C. parapsilosis* isolates have highly homozygous genomes, with approximately one heterozygous Single Nucleotide Polymorphism (SNP) per 15 kb (Butler *et al.* 2009) and some Copy Number Variations (CNVs), which may have resulted from recombination (Pryszcz *et al.* 2013). In contrast, *C. metapsilosis* isolates have very heterozygous genomes, and most likely arose from hybridization between two related species (Pryszcz *et al.* 2015). The first *C. orthopsilosis* genome sequence, with a size of ∼12.66 Mb, was from a highly homozygous isolate (Riccombeni *et al.* 2012). Subsequent analysis showed that the genomes of most *C. orthopsilosis* isolates have high levels of heterozygosity. For example, the average number of SNPs is approximately 1 in 32 bp in *C. orthopsilosis* (Schröder *et al.* 2016) compared to 1 in 330 in *C. albicans* and 1 in 576 bp in *Lodderomyces elongisporus* (Butler *et al.* 2009). *C. orthopsilosis* isolates probably originated from at least four separate hybridization events between the same pair of parental species (Pryszcz *et al.* 2014; Schröder *et al.* 2016).

Here, we investigate the correlation between genotype and phenotype in 28 isolates of *C. orthopsilosis*, using cosine analysis. We identified >60 high-impact variants that are potentially correlated with phenotype. We implemented CRISPR/Cas9-based gene editing to confirm the effect of one high-impact variant on caffeine sensitivity.

## Materials and methods

### Strains and growth conditions

*C. orthopsilosis* strains (Table S1) were stored at -80° in media containing 15% glycerol before being subcultured onto YPD agar medium (1% yeast extract, 2% peptone, 2% glucose and 2% agar) and incubated at 30°. For phenotype analysis, isolates were inoculated as 2x2 arrays (two independent cultures with one technical replicate of each) into 200 μl of YPD broth (1% yeast extract, 2% peptone, 2% glucose) in 96-well plates and incubated at 30° for 24 h. Stocks were diluted in 96-well plates containing 200 μl of water by dunking a 12x8 pin bolt replicator (V&P Scientific) three times in the culture and then transferring it to the water. The diluted cultures were then pinned onto solid agar media and incubated at 30° for 72 h. For phenotyping measurements, plates were photographed and growth was compared to YPD using SGAtools (Wagih *et al.* 2013). All phenotype images and growth scores are available at FigShare (File S1). SGAtools was designed to analyze synthetic genetic interactions and assumes that average growth on a plate does not vary. This was not true for several media, where many strains grew poorly. We therefore compared the growth of each strain on the test media to the growth of the same strain on YPD, using the raw data extracted from SGAtools.

For each strain in each analyzed growth condition, the SGAtools scores were converted to a binary score where 0 represents everything with a growth ratio above 0.45 (no, or minor, growth defect) and 1 represents scores below or equal to 0.45 (major growth defect). The SGAtools scores are normally distributed with a mean of 0.99, a median of 1.04, a 1^st^ quartile of 0.76 and a 3^rd^ quartile of 1.25. The cut-off of 0.45 was manually chosen after inspection of the phenotype plates. This score corresponds to a major growth defect.

### Correlation between genotype and phenotype

Variants in each genome relative to the reference *C. orthopsilosis* 90-125 were identified as previously described (Schröder *et al.* 2016). Insertions, deletions and single nucleotide variants were identified using the GATK HaplotypeCaller (McKenna *et al.* 2010) with -nct 42 threads. Variants with mapping quality scores <40 and read depth <15 were removed. Variants from all strains were merged using GATK CombineVariants and are available at FigShare (File S2). Variants were functionally annotated with SnpEff (Cingolani *et al.* 2012), using the reference genome sequence from *C. orthopsilosis* 90-125 and gene annotation from the Candida Gene Order Browser (Fitzpatrick *et al.* 2010; Maguire *et al.* 2013). Variants with a high putative impact on a gene, as identified by SnpEff, were retained (available at FigShare, File S2). All high impact variants in each strain were converted to a binary score, with 1 representing a heterozygous or homozygous variant, and 0 representing absence of the variant. The correlation of the phenotype and genotype binary scores was then assessed using a cosine similarity measure in R (Gentleman *et al.* 2004). 1415 high impact variants with cosine similarity measures above or equal to 0.85 were retained at this stage. Variants that had an adjacent high impact variant within 10 bases or that were present in more than 20 alleles were subsequently excluded from further analysis, because they most likely result from poor alignment. Step 1 reduced the number of variants to 423, and step 2 reduced the variant number to 65 (Table S3). All remaining 65 variants were manually inspected in a local genome browser.

### Editing ZCF29 with CRISPR-Cas9

A 20 bp protospacer sequence (guide RNA) targeting *C. orthopsilosis ZCF29* (*CORT0G04310*) was designed using the Eukaryotic Pathogen CRISPR guide RNA Design Tool (Peng and Tarleton 2015). The guide RNA was generated by annealing of two short oligos (gCoZCF29_TOP/BOT, Table S2) and the insert was cloned into the SapI-digested pCP-tRNA plasmid to generate plasmid pCP-tRNA-ZCF29, as previously described (Lombardi *et al.* 2019). The repair template (RT-CoZCF29) carrying the desired stop codon and 1 synonymous SNP to disrupt the PAM sequence was generated by primer extension (rCoZCF29_TOP/BOT, Table S2) using the ExTaq DNA polymerase (TaKaRa Bio, USA). *C. orthopsilosis* strains 1825 and 151 were transformed with 5 μg pCP-tRNA-ZCF29 and 25 μl of unpurified RT-CoZCF29 using the method previously described for *C. parapsilosis* (Holland *et al.* 2014). Transformants were selected on YPD agar plates containing 200 μg/ml nourseothricin (NTC), incubated at 30° for 48 h. Two NTC resistant transformants for each strain were sequenced using sCoZCF29_FWD/pCoZCF29_REV. The pCP-tRNA-ZCF29 plasmid was cured by growing the cells in the absence of selection on YPD until they could not grow anymore in the presence of NTC.

### Galleria mellonella infection

On delivery, *Galleria mellonella* larvae (Livefood UK Ltd.) were kept at 15° for 1 week and used for experimentation within a month. *C. orthopsilosis* strains were grown overnight in a shaking incubator at 30° in 5 ml of YPD medium. Overnight cultures were collected by centrifugation and washed twice with Phosphate Buffered Saline (PBS, Oxoid) at 13000 rpm at room temperature for 1 min. Cells were washed twice in 1 ml of PBS and resuspended in 1 ml of PBS. Using a hemocytometer, the concentration of the inocula were calculated and adjusted to 5 × 10^7^ cells/ml. Twenty larvae, similar in size and weight, were used to analyze the virulence of each fungal strain. Larvae were injected with 10 µl of the diluted strains through the last left proleg, using insulin syringes. Untreated larvae and larvae injected with PBS were used as negative controls to assess the general viability and the effect of injection, respectively. After inoculation, larvae were kept at 30° in the dark. The viability of larvae was monitored every 24 hr, for four days. Virulence was analyzed by comparing the survival curves over time (by Kaplan-Meier estimate with a log-rank test). The statistical analyses were performed using the IBM SPSS Statistics 24 software package.

### Data availability

All strains and plasmid are available upon request. Images and data analysis files (FileS1), VCF files (FileS2) and supplementary tables are available on FigShare. Table S1 contains a list of strains used, Table S2 contains the sequences of oligonucleotides used, Table S3 contains the raw and filtered high impact variants that correlate with phenotype data. Supplemental material available at Figshare: https://doi.org/10.25387/g3.8864960.

## Results

### Phenotype analysis of C. orthopsilosis

**Figure 1:**
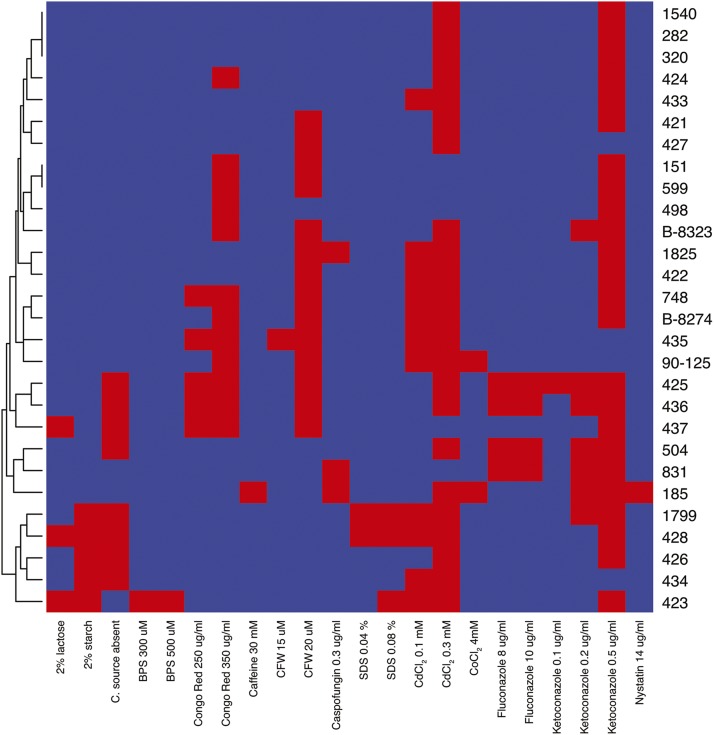
Phenotype analysis of *C. orthopsilosis* isolates. Growth defects of 28 *C. orthopsilosis* strains (y-axis) on solid media (x-axis). Chemicals were added to YPD at the indicated concentrations. All media contained 2% glucose, except where substituted with 2% lactose or 2% starch. Growth scores were calculated by comparing the average growth of at least 4 replicates on each condition to growth on YPD, using SGAtools. Scores ≤0.45 (decreased growth) are shown in red, and all other scores are in blue. Only conditions for which a growth defect is observed for at least one strain are shown. Scores are visualized using the gplots package in R (Warnes *et al.* 2016).

Most *C. orthopsilosis* isolates fall into four or five clades that arose from independent hybridization events between related, but not identical parents termed A and B (Schröder *et al.* 2016). Two isolates (strains 90-125 and 428) have highly homozygous genomes and represent parent A. The remaining isolates demonstrate varying levels of heterozygosity (Schröder *et al.* 2016). We examined the growth characteristics of the 28 sequenced *C. orthopsilosis* isolates, representing all clades, in 60 different conditions, including alternative carbon sources, heavy metals, and exposure to antifungal drugs. Cultures were pinned to solid media including at least four replicates per strain, and growth was scored after 72 h growth using SGAtools (Wagih *et al.* 2013). SGAtools calculates colony size and averages all replicates. We compared the growth of *C. orthopsilosis* isolates on various media to growth on YPD, a rich media. Colonies with scores ≤0.45 (*i.e.*, colony size was less than or equal to 45% on the test condition relative to growth on YPD) were assumed to have a moderate to severe growth defect in the tested conditions. Figure 1 shows the growth defects of all strains in 22 different conditions. All images and scores are available in File S1.

Some phenotypes are specific to one isolate or a small group of isolates. For example, strain 185 (clade 3) grows less well than all other strains on 14 μg/ml nystatin, whereas strains 428 and 1799 grow poorly in the presence of SDS (Figure 1).

### Correlating genotype and phenotype

We previously reported the genome sequences of the 28 *C. orthopsilosis* isolates (Riccombeni *et al.* 2012; Schröder *et al.* 2016). Homozygous and heterozygous single nucleotide polymorphisms and insertions and deletions relative to the reference genome *C. orthopsilosis* 90-125 (Riccombeni *et al.* 2012) were identified (Schröder *et al.* 2016). Here, we attempt to correlate these variants with specific phenotypes.

**Figure 2:**
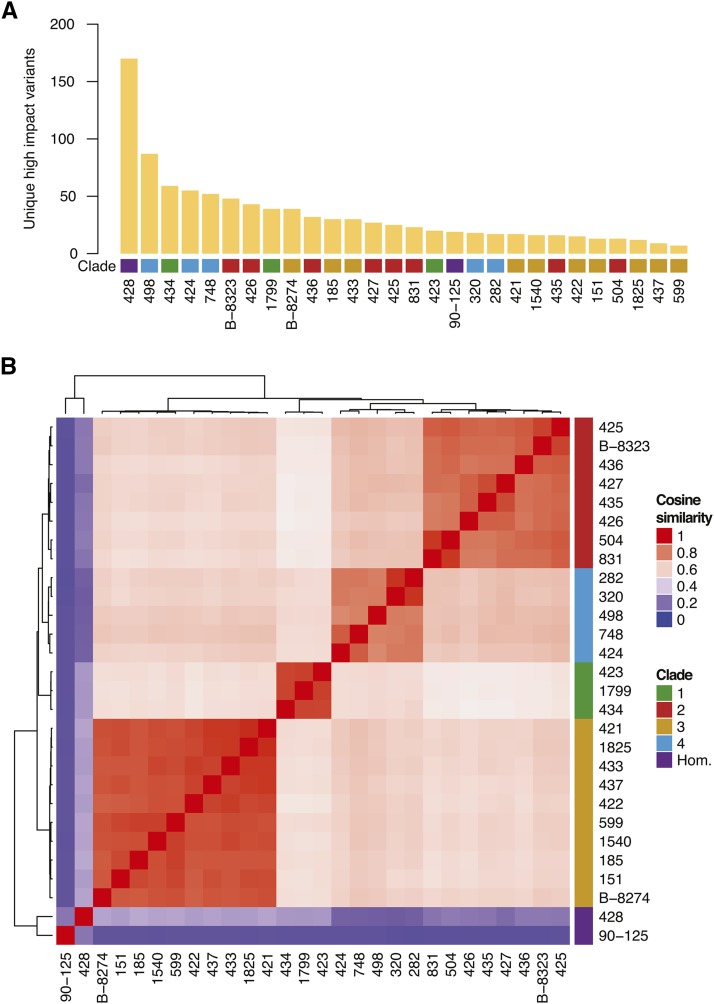
High impact variants are shared by isolates within a clade. (A) Barplot illustrating the number of unique high impact variants per strain. Clade designation of each strain is represented by colors, Clade 1 (green), Clade 2 (red), Clade 3 (orange), Clade 4 (blue) and Homozygous (purple). (B) Comparison of high-impact variants for each strain using cosine similarity measure. A cosine similarity value of 1, in red, represents high correlation and a value of 0, in blue, represents no correlation. High-impact variants are shared by clade members. There are few variants in the two homozygous strains 428 and 90-125.

Putative high impact variants were identified using SnpEff, which predicts coding effects of variants (Cingolani *et al.* 2012). High impact variants are defined as those that cause frameshifts, introduce stop codons, lose start codons, or lie within splice acceptor or splice donor sites. A total of 6679 high impact variants were identified in 28 *C. orthopsilosis* isolates relative to the reference annotation. The number of unique high-impact variants varies between strains (Figure 2A), with isolate 599 having the least and isolate 428 the most. Some variants are shared by isolates within a clade (Figure 2B).

Population structure can interfere with the identification of variants associated with phenotypes, especially in asexual species where the structure can result from clonal reproduction (Taylor *et al.* 2017). Some of these issues can be addressed using Linear Mixed Models (Peter *et al.* 2018). Because we are characterizing a small number of very heterozygous isolates, we decided to use simple cosine measurements to investigate relationships between genotype and phenotype. The presence of each variant in each strain was represented using a binary score, where 1 represents the presence of a heterozygous or homozygous variant, and 0 represents absence of the variant. Growth phenotypes were also scored as either 1 (representing a growth defect, *i.e.*, a score ≤0.45), or 0 (representing no growth defect). Correlation between growth scores and high impact variants was then determined using cosine similarity. The cosine similarity indicates how alike two vectors are to each other. In our case, one vector contains information about the presence or absence of a variant across all strains, and the second vector records the presence or absence of a growth defect across all strains. 65 variants with a cosine similarity value >0.85 were identified following filtering, as described in Methods (Table S3).

The majority of the identified variants that potentially correlate with phenotype result in predicted frameshifts. Some of these may be artifacts resulting from repetitive regions in the sequence, or genome assembly errors in the reference genome. We therefore restricted our analysis to highly supported SNPs that result in gain or loss of stop codons, loss of start codons, or variants that affect splicing. There were 19 variants of these types (Table 1). Because many isolates have more than one variant that correlates with the same phenotype, it is difficult to identify the causative allele using this method alone.

**Table 1:** List of SNPs correlated with phenotype. See Table S3 for full list of variants

*Isolate*	*Media*	*Gene ID*	*Gene common*	*Impact*	*Copy No*	*Mutation^1^*
185	Caffeine 30 mM, Nystatin 14 μg/ml	B03950		Stop gained	Het	Arg182*
		E01960	*YND1*	Stop gained	Het	Gln456*
		F01150	*SFL2*	Stop gained	Het	Gln289*
		H02450	*CDC39*	Stop gained	Het	Gln1194*
		B03390	*SLD1*	Start lost	Het	Met1?
		C04260	*TSA1*	Stop gained	Hom	Leu135*
		G04310	*ZCF29*	Stop gained	Hom	Gln814*
425	Ketoconazole 0.1 μg/ml	C01460	*RAD50*	Stop gained	Het	Tyr599*
		D06060	*RGD2*	Stop gained	Het	Gln581*
		D01450	*RVS162*	Stop gained	Het	Tyr56*
		E03200		Stop gained	Het	Leu32*
		H01350		Stop gained	Het	Trp288*
425, 426, 436, 504, 831	Fluconazole 8 μg/ml and 10 μg/ml	A02170	*AIP1*	Stop gained	Het	Lys4*
423	BPS 300 μM and 500 μM	E04360		Stop gained	Het	Arg143*
435	Calcofluor White 15 μM	B06120	*APM4*	Stop gained	Het	Tyr20*
		H00190	*FRP2*	Stop gained	Het	Ser540*
1799, 423, 434, 428	SDS 0.08%, 2% starch	B11130	*YOR296W*	Start lost	Hom	Met1?
		B11240		Stop gained	Hom	Trp2*
		E01950		Stop gained	Het (428 Hom)	Lys215*

1 * = Non-synonymous SNP leading to stop codon at indicated amino acid position, Met1? = Methionine (start codon) at amino acid position 1 is lost.

**Figure 3:**
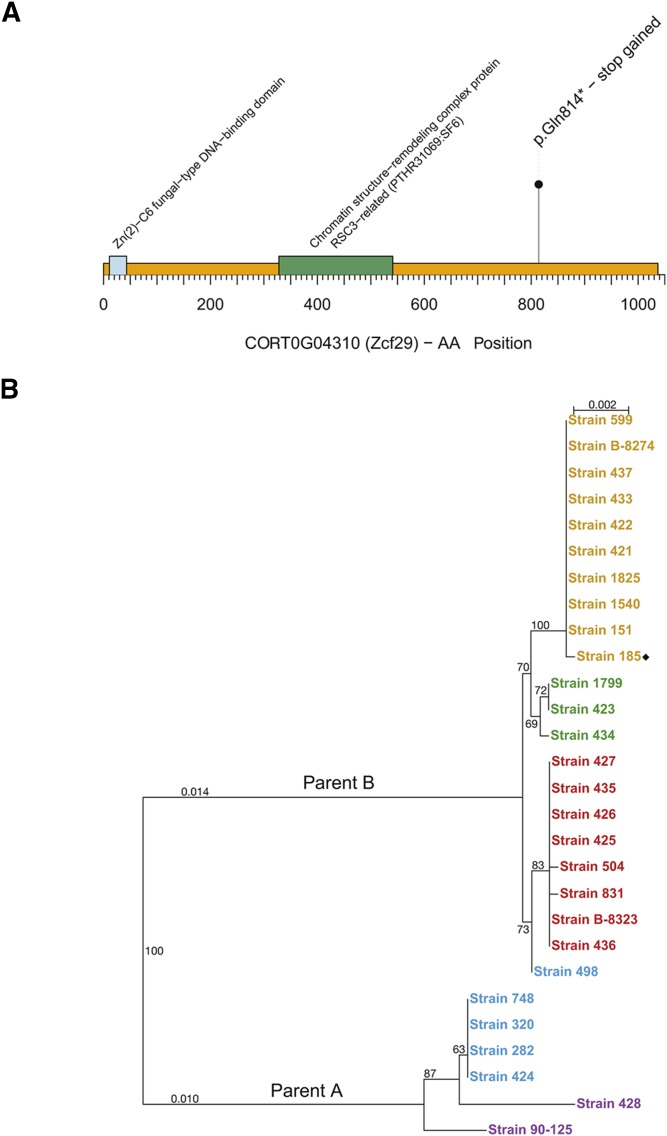
Overview of Zcf29. (A) The presence of a premature stop codon in strain 185 is represented by the lollipop. The protein domains are shown as blue and green squares. AA = Amino acid. (B) Phylogenetic relationship of all *ZCF29* sequences from *C. orthopsilosis* isolates. Most *C. orthopsilosis* strains are derived from hybridization between two parents (A and B). The phylogenetic tree shows that most strains retain both alleles of *ZCF29* from the B parent after hybridization. Strain 185 (premature stop codon) is highlighted with a rhombus. Strains are color-coded based on their clade of origin (Clade 1 in green, Clade 2 in red, Clade 3 in orange, Clade 4 in blue, homozygous strains in purple).

Strain 185 (CAS08-0185, (Schröder *et al.* 2016)) is sensitive to growth on caffeine, nystatin, ketoconazole, caspofungin, cobalt chloride and cadmium chloride (Figure 1). Cosine analysis suggested that sensitivity to caffeine and/or nystatin may be associated with variants that are specific to this isolate. These include four heterozygous SNPs resulting in premature stop codons, one heterozygous SNP resulting in a premature start, and two homozygous SNPs resulting in premature stops in both alleles at two loci (*TSA1* and *ZCF29*) (Table 1). *TSA1* encodes a predicted alkyl hydroperoxide peroxidase C, that is likely to be associated with oxidative stress signaling (Urban *et al.* 2005), whereas deleting *ZCF29* in *C. albicans* results in increased sensitivity to caffeine (Homann *et al.* 2009). The C to T SNP in *ZCF29* in *C. orthopsilosis* isolate 185 replaces glutamine 814 with a stop codon (Q814*), resulting in a protein that is 222 amino acids shorter than the wild type (Figure 3). The *ZCF29* gene was therefore chosen for additional analysis.

Because most *C. orthopsilosis* isolates are hybrids of two parents (called A and B) (Schröder *et al.* 2016), the *ZCF29* alleles in sample 185 could be derived from either parent. Phylogenetic analysis of *ZCF29* from all isolates (Figure 3) shows that in sample 185 and 20 other isolates, both alleles are derived from parent B. In another 6 isolates both alleles are derived from parent A. One isolate (sample 498) is unusual - the 5′ end of the gene suggests that one allele is derived from parent A and one from parent B, but the remaining of the gene has undergone LOH so that both alleles resemble parent B.

### Editing ZCF29 in C. orthopsilosis

To test if the homozygous stop codon variant in *ZCF29* in sample 185 would confer sensitivity to caffeine, nystatin or other drugs in other genetic backgrounds, we used the CRISPR-Cas9 technology to introduce the variant into *C. orthopsilosis* isolates 151 (CAS08-0151) and 1825 (CAS10-1825), which belong to the same clade as sample 185 (Schröder *et al.* 2016) but are not sensitive to caffeine or nystatin (Figure 1).

**Figure 4:**
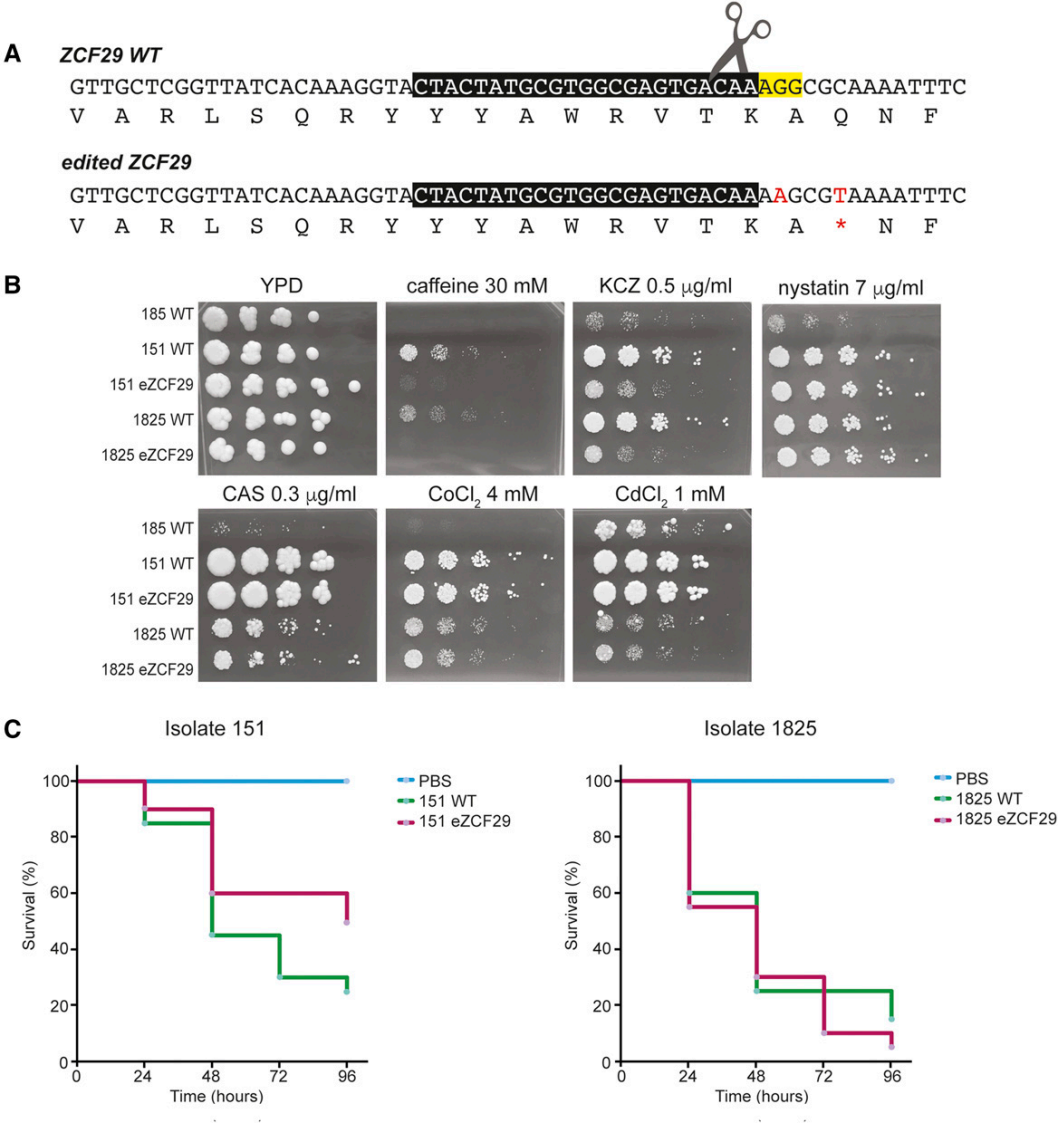
Introducing stop codons in *ZCF29* in *C. orthopsilosis* confers sensitivity to caffeine and ketoconazole, and does not affect virulence. (A) CRISPR-editing was used to introduce the homozygous variant in *ZCF29* in sample 185 into strains 151 and 1825. After the Cas9-induced double-strand break (scissors), a repair template was used to introduce one synonymous SNP to disrupt the PAM (in yellow) and the C > T SNP resulting in the premature stop codon. The guide sequence is boxed in black. The SNPs introduced in the mutated sequence are highlighted in red. (B) Wild type and *ZCF29* edited versions of strains 151 and 1825, and the original strain 185, were grown in YPD overnight and diluted in PBS before plating on YPD agar plates containing nystatin, caffeine, ketoconazole (KCZ), caspofungin (CAS), cobalt chloride (CoCl_2_), or cadmium chloride (CdCl_2_) at the indicated concentrations. Plates were photographed after 72h at 30°C. Strains with an introduced premature stop codon in *ZCF29* showed reduced growth on caffeine and ketoconazole compared to parental strains. (C) Kaplan-Meier curves illustrating the survival over time of *G. mellonella* larvae injected with *C. orthopsilosis* strains. Twenty larvae were used per strain. Log-rank test results comparing wild type strains (in green) and *ZCF29* edited strains (in pink) show no significant differences between them (*P* = 0.109 in isolate 151 and *P* = 0.446 in isolate 1825).

A guide RNA was expressed on a plasmid between a tRNA and a ribozyme (Lombardi *et al.* 2019) generating plasmid pCP-tRNA-CoZCF29, and was transformed together with a repair template RT-CoZCF29, designed to introduce a stop codon at amino acid 814 (Q814*), reproducing the polymorphism seen in strain 185. A synonymous SNP was also introduced to destroy the PAM site targeted by the guide RNA (Figure 4A). The edited mutants (151eZCF29 and 1825 eZCF29) were confirmed by sequencing.

### Editing ZCF29 confers sensitivity to caffeine

Cosine analysis suggested that variants in strain 185 correlated with sensitivity to caffeine and nystatin (Table 1). However, strain 185 is also sensitive to ketoconazole, caspofungin, cobalt chloride, and cadmium chloride (Figure 1). We therefore tested the growth of the edited strains in all conditions. Strains with an introduced premature stop codon in *ZCF29* showed reduced growth in caffeine and ketoconazole (0.5 μg/ml), compared to parental strains (Figure 4B). No differences in growth between the edited strains and their parental wild type strains were observed in the presence of nystatin, caspofungin, cobalt chloride, or cadmium chloride (Figure 4B).

### Editing ZCF29 does not alter virulence of C. orthopsilosis

In *C. albicans*, mutating *ZCF29* results in reduced filamentation and decreased colonization of mouse organs (Vandeputte *et al.* 2011). To test if disrupting *ZCF29* reduces the pathogenicity of *C. orthopsilosis*, we compared the virulence of the edited strains to their wild-type strains in the model host *G. mellonella* (Figure 4C). The virulence of the edited strains is not significantly different from their respective wild type parental strains (log-rank test, Figure 4C).

## Discussion

Identification of specific polymorphisms associated with phenotypes is challenging, and usually requires linkage analysis, or combining linkage studies with genome sequencing. However, it is difficult to carry out association studies in asexual species. In this study, we identified 65 high impact variants in the asexual yeast *C. orthopsilosis* that are linked to specific phenotypes. 19 are SNPs that likely represent high confidence variants.

We validated our approach by testing the effect of one variant which introduces a premature stop codon in both alleles of the transcription factor *ZCF29* and is present only in *C. orthopsilosis* strain 185. This variant correlated with decreased growth relative to other strains when caffeine or nystatin is present in the media. We found that we could recapitulate the caffeine sensitivity phenotype by introducing the variant into *ZCF29* in two different *C. orthopsilosis* isolates. Nystatin sensitivity may be associated with other variants in this isolate, such as the homozygous premature stop in *TSA1*, or heterozygous stops in 5 other alleles (Table 1). However, we found that editing *ZCF29* also reduced ketoconazole sensitivity, although we did not identify a significant correlation between ketoconazole sensitivity and the variant (Table 1). It is therefore likely that ketoconazole sensitivity is caused by many different variants in different *C. orthopsilosis* isolates.

Zcf29 belongs to the Zn(II)2Cys6 zinc cluster family of transcription factors in fungi. In *C. albicans*, mutations in *ZCF29* result in sensitivity to the TOR (target of rapamycin) inhibitor beauvericin, to caffeine and to the oxidative stress inducer menadione (Homann *et al.* 2009; Vandeputte *et al.* 2011; Shekhar-Guturja *et al.* 2016). The role of *ZCF29* in caffeine sensitivity is conserved between *C. albicans* and *C. orthopsilosis*. *C. albicans* mutants are also unable to filament at 42° or in serum containing media or to colonize mouse kidneys (Vandeputte *et al.* 2011). At least some of these phenotypes result from regulation of expression of multidrug transporter genes (Shekhar-Guturja *et al.* 2016).

The presence of stop codons in *ZCF29* in isolates belonging to one *C. albicans* clade has been associated with reduced virulence in this species (Ropars *et al.* 2018). Editing *ZCF29* in *C. orthopsilosis* did not have any effect on virulence. However, the stop codon was introduced after the predicted DNA binding and chromatin structure-remodelling domains (Figure 3). It is therefore possible that the truncated protein retains some function. In addition, we monitored virulence using the mini-host *G. mellonella*. There is some evidence that the response of *Galleria* to infection can mimic the response of the mammalian host. For example, in a recent study, 10 of 18 *C. albicans* deletion strains tested had similar effects on fungal load in mice and in *G. mellonella* larvae (Amorim-Vaz *et al.* 2015). Deleting *C. albicans SPT20* also reduces virulence in both mice and larvae (Tan *et al.* 2014). However, other studies found inconsistencies. For example, Frenkel *et al.* (2016) found that a *C. albicans* strain isolated from the vaginal tract (M39) was highly virulent in mice, but avirulent in the insect model. Our interpretation that *C. orthopsilosis ZCF29* does not impact virulence should therefore be treated with some caution.

We have shown that it is possible to use cosine similarity analysis to link high impact variants with specific phenotypes in *C. orthopsilosis*. However, we have explored only a small amount of the genetic variation in the species. The high number of homozygous and heterozygous polymorphisms in hybrid species such as *C. orthopsilosis* (one SNP per ∼32 bp), and in particular the high number of apparent indels, CNVs and frameshifts, makes identifying phenotypic-genotypic associations particularly difficult. The approach may therefore be more successful in more homozygous species such as *C. parapsilosis* (one SNP per 15 kb).
